# Down-regulation of miR-210-3p encourages chemotherapy resistance of renal cell carcinoma via modulating ABCC1

**DOI:** 10.1186/s13578-018-0209-3

**Published:** 2018-02-07

**Authors:** Songchao Li, Jinjian Yang, Jun Wang, Wansheng Gao, Yafei Ding, Yinghui Ding, Zhankui Jia

**Affiliations:** 1 Department of Urology, The First Affiliated Hospital, Zhengzhou University, No 1 Jianshe East Rd., Zhengzhou, 450052 People’s Republic of China; 2 Urological Institute of Henan, Zhengzhou, 450052 Henan Province People’s Republic of China

**Keywords:** Renal cell carcinoma, Multi-drug resistance, MiR-210-3p, ABCC1, MDR-1

## Abstract

**Background:**

ATP-binding cassette transporter super-family including ABCC1 and MDR-1 were involved in multi-drug resistance (MDR) of renal cell carcinoma (RCC) patients. Several miRNAs were confirmed to promote the MDR and the survival of tumor cells.

**Methods:**

The RCC cell lines Caki-2 with vinblastine-resistant (Caki-2/VBL) or doxorubicin-resistant (Caki-2/DOX) were constructed, respectively. The expressions of miR-210-3p, ABCC1 and MDR-1 protein were determined by qRT-PCR and Western blot assays. The viability of RCC cells was assessed by MTT assay. The regulatory relationship between miR-210-3p and ABCC1 was analyzed by Dual Luciferase assay. The effect of miR-210-3p in vivo was investigated with a tumor xenograft model in mice.

**Results:**

MiR-210-3p expression was observed to significantly decrease in Caki-2/VBL and Caki-2/DOX cells. Meanwhile, ABCC1 and MDR-1 were significantly increased in Caki-2/VBL and Caki-2/DOX cells. ABCC1 was a novel target of miR-210-3p and negatively regulated by miR-210-3p. And miR-210-3p improved drug-sensitivity of RCC cells. Down-regulation of ABCC1 could reverse the effect of miR-210-3p knockdown on the drug-resistance and the level of MDR-1 in drug-sensitive RCC cells.

**Conclusion:**

We confirmed that down-regulation of miR-210-3p increased ABCC1 expression, thereby enhancing the MRP-1-mediated multidrug resistance of RCC cells.

## Background

Renal cell carcinoma (RCC) is one of the most lethal urologic malignancies worldwide with significant morbidity, mortality and poor prognosis [1]. The surgical therapy, including radical resection or nephron-sparing surgery, was commonly used as the preferred method for RCC. For those with advanced or recurring RCC patients, chemotherapy is the mainstream method for RCC in clinic, but it has unsatisfactory results in RCC patients [2]. The main reason for chemotherapy failure is that RCC cells develop multidrug resistance (MDR) to chemotherapy agents, such as vinblastine and doxorubicin [3]. MDR expanded the ability of RCC cells to resist the cytotoxicity induced by chemotherapy agents, which was also accurately regulated by non-coding RNAs, proteins and signaling pathways [4]. The exploration of MDR mechanisms in RCC has become a new research direction in this field.

RCC patient with insensitivity to conventional chemotherapy agents may attribute to the intrinsic or acquired multi-drug resistance. ABCC1 and MDR-1, two numbers of ATP-binding cassette transporter super-family related to multi-drug resistance, were documented to increase in RCC patients and served as the efflux pumps to promote chemotherapeutic drugs out of cancer cells via the assistance of ATPase activity [5, 6]. The expression of ABCC1 and MDR-1 could act as the MDR markers in RCC [6]. However, the relative contributions and causative roles of ABCC1 and MDR-1 in MDR of RCC cells have not been completely clarified.

MicroRNAs (miRNAs), a class of non-coding RNAs with the length of 18-25nt, are implicated in various fundamental biological processes and cancer pathological processes [7] through binding with 3′UTR of target mRNAs, thereby causing the inhibition of translation and the degradation of mRNA, eventually to modulate gene expression at the post-transcriptional level [8]. More and more evidences have indicated that the aberrant miRNA expression is related to drug resistance/sensitivity and pathology of RCC [9, 10]. MiR-210-3p was reported to be depleted by CRISPR/Cas9 to promote tumorigenesis through TWIST1 revival in RCC [11]. Moreover, miR-210-3p was predicted to have the binding site on the 3′UTR of ABCC1. Hence, we hypothesized that miR-210-3p was involved in the underlying mechanism of MDR in RCC.

## Methods

### Cell culture and induction of drug-resistant cell lines

Caki-2 cells, a human RCC cell line, were purchased from American Type Culture Collection (ATCC; Manassas, VA, USA) and were cultured in the McCoy’s 5A medium (Thermo Fisher scientific, Massachusetts, USA) supplied with 10% FBS and 100 µg/ml double-antibody at 37 °C with the humidified 5% CO_2_. Caki-2/DOX (doxorubicin-resistant) and Caki-2/VBL (vinblastine-resistant) cells, the drug-resistant RCC cell lines, were constructed via Caki-2 cell lines (their independent parental cell lines) being exposed to the IC_50_ concentration of DOX and VBL for 3 months, and then exposed to tenfold higher dose of IC_50_ for 6 months with the same cultural conditions as Caki-2 cell lines [12].

### Cell transfection

The RCC cell lines (Caki-2, Caki-2/DOX, and Caki-2/VBL) were seeded in the 6-well plates with the density of 2 × 10^5^ cells/ml, and then maintained for 24 h. Caki-2 cells were transfected with miR-210-3p mimic/pre-NC (20 nM) or miR-210-3p inhibitor (50 nM) + si-ABCC1/si-control (20 nM) using Lipofectamine 2000 (Invitrogen, USA) following the manufacturer’s protocol. Caki-2/DOX and Caki-2/VBL cells were transfected with miR-210-3p mimic/NC or miR-210-3p mimic + pcDNA-ABCC1/pcDNA by Lipofectamine 2000 (Invitrogen). The transfected RCC cells were maintained for 48 h, followed by harvested for the next experiments. The detailed sequence information was as follows: miR-210-3p mimic, 5-CUGUGCGUGUGACAGCGGGUGA-3; miR-210-3p inhibitor, 5-UCAGCCGCUGUCACACGCACAG-3; si-ABCC1, 5-GUUCCAAGGUGGAUGCGAATT-3. ABCC1 overexpression construct (pcDNA-ABCC1) was synthetized by Guangzhou RiboBio Co., Ltd (Guangzhou, China). The ABCC1 sequence was amplified with forward (F, 5-GTCGACACCATGGCCTGCTATTGC-3) and reverse (R, 5-GATGGATCCGCAGCAGAATGCCCAG-3) primers. After sequence validation, the sequences were subcloned into pcDNA3.1 vector.

### Quantitative real-time PCR

The levels of miR-210-3p expression and ABCC1 mRNA expression were determined by quantitative real-time PCR (qRT-PCR). Total RNA was extracted from RCC cell lines using TRIzol (Invitrogen). The extracted RNAs were reverse transcribed to complementary DNA with the PrimeScript^®^ RT reagent kit (TaKaRa). The levels of miR-210-3p expression and ABCC1 mRNA expression were quantified by SYBR^®^ Premix DimerEraser kit (TaKaRa) with 7500 Fast Real-Time PCR System in the ABI Prism 7500 (Applied Biosystems). U6 was used as the internal control for miR-210-3p, and GAPDH acted as the internal control for ABCC1. Comparative CT method, 2^−ΔΔCt^, serves as the calculation method of relative gene expression. The primer sequences used in qPCR were as follows: miR-210-3p, forward 5-GTGCAGGGTCCGAGGT-3, reverse 5-TATCTGTGCGTGTGACAGCGGCT-3; MDR1, forward 5-CCCATCATTGCAATAGCAGG-3, reverse 5-TGTTCAAACTTCTGCTCCTGA-3; ABCC1, forward 5-ATGTCACGTGGAATACCAGC-3, reverse 5-GAAGACTGAACTCCCTTCCT-3; U6, forward 5-CTCGCTTCGGCAGCACA-3, reverse 5-AACGCTTCACGAATTTGCGT-3.

### Western blotting

The levels of ABCC1 and MDR-1 protein were assessed by Western blot assays in accordance with previous report [13]. Total protein from RCC cell lines was extracted with RIPA lysis buffer and then separated by SDS-PAGE on 10% acrylamide gels, followed by transferred into PVDF membrane. Afterwards, the membrane was incubated with primary antibodies against ABCC1 (1:1000 dilution, Abcam, Cat. no. ab99531), MDR-1 (1:500 dilution, Abcam, Cat. no. ab129450) and β-actin (1:3000 dilution, Abcam, Cat. no. ab8226) (4 °C overnight) and then maintained with HRP-conjugated secondary antibody for 1 h. Protein bands were visualized with Amersham ECL Western blotting detection reagents (GE Healthcare, Piscataway, NJ, USA).

### Cell viability

For drug-resistant analysis, the cell viability assays were performed. RCC cells (2 × 10^4^ cells/well) were planted in 96-well plates and cultured at 37 °C with a 5% CO_2_ humidified atmosphere for 24 h. Afterwards, DOX with various concentrations (0, 10, 50, 100, 200, 400 μg/ml and 0, 1, 2, 3, 4, 5 μg/ml) were respectively administrated to Caki-2/DOX and Caki-2 cell culture, and VBL at various concentrations (0, 10, 50, 100, 200, 300 and 0, 1, 2, 3, 4, 5 μg/ml) were respectively administrated to Caki-2/VBL and Caki-2 cell culture for 24 h of incubation. The MTT assays were applied to analyze the viabilities of each RCC cell line in accordance with previous report [14]. MTT solutions (20µl, 5 mg/ml) was added to each well for 4 h at 37 °C.

### Dual Luciferase assay

The bind site of miR-210-3p and ABCC1 were predicted and the wild type (WT) and mutant (Mut; the bind site was mutant) fragment of ABCC1 was shown in Fig. 3a. Two fragments were amplified by PCR using the primers: for the WT segment, 5′-AATTAGATCTAAAGAAAAGCGAGAGCAGCA-3′ (forward) and 5′-AATTAGATCTGCTCTCTGGGTTTGAAGTCG-3′ (reverse); for the Mut segment, 5′-AATTAGATCTGCTGTGAAGCACACGGAGAG-3′ (forward) and 5′-AATTAGATCTCAGACATTCGCGGTCAGAGA-3′ (reverse). Two ABCC1 fragments were respectively cloned into the downstream of the luciferase gene of pGL3 Luciferase miRNA Target Expression Vector (Promega, Madison, WI, USA) to synthesize the recombinant reporter vector named pGL3-ABCC1-WT and pGL3-ABCC1-Mut. The reporter vector pGL3-ABCC1-WT/pGL3-ABCC1-Mut and miR-210-3p inhibitor/NC were co-transfected into Caki-2 cells using Lipofectamine 2000 (Invitrogen). The reporter vector pGL3-ABCC1-WT/pGL3-ABCC1-Mut and miR-210-3p mimic/pre-NC were co-transfected into Caki-2/DOX and Caki-2/VBL cells using Lipofectamine 2000 (Invitrogen). After positive lysis of the cells, multimode detector with the Dual-Luciferase Reporter Assay System (Promega) was used to evaluate the activities of luciferase.

### Xenograft model

In order to analyze the effects of miR-210-3p on drug-resistant renal tumor growth in vivo, a nude mouse tumor xenograft model was established. The present study was approved by Animal Care and Experimentation Committee of The First Affiliated Hospital of Zhengzhou University. Nude mice were transplanted subcutaneously with 2.5 × 10^6^ Caki-2/DOX cells with/without miR-210-3p over-expression into the right flank [pre-NC group (n = 8) and miR-210-3p mimic group (n = 8)]. After 10 days, the mice of two groups were treated with DOX (2 mg/kg/day) via intraperitoneal injections. Every 3 days, the length (L) and width (W) of tumor in mice were measured, and the tumor volume was calculated using the following equation: (L × W^2^)/2. After 30 days, the mice were killed and tumor tissues were removed for the following study.

In the following experiment, Mice were transplanted subcutaneously with 2.5 × 10^6^ Caki-2 cells with/without miR-210-3p knockdown into the right flank [NC group (n = 8) and miR-210-3p inhibitor group (n = 8)]. After 10 days, the mice of two groups were treated with DOX (2 mg/kg/day) via intraperitoneal injections. Every 3 days, the length (L) and width (W) of tumor in mice were measured, and the tumor volume was calculated using the following equation: (L × W^2^)/2. After 30 days, the mice were killed and tumor tissues were removed for the following study.

### Statistical analysis

All data from three independent repeated experiments were exhibited as the mean ± SD and statistically analyzed with Student’s t test for single comparison between two groups and one-way ANOVA for comparison of multiple groups on SPSS 17.0 (SPSS Inc., Chicago, IL, USA). A value of P less than 0.05 was considered statistically significant.

## Results

### The expression of miR-210-3p was decreased and the levels of ASCC1 and MDR-1 were increased in drug-resistant RCC cells

**Fig. 1:**
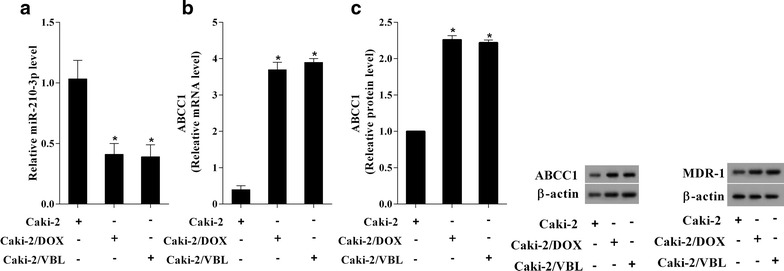
The expression of miR-210-3p, ABCC1 and MDR-1 in drug-sensitive and drug-resistant RCC cells. The RCC cell line Caki-2 with vinblastine-resistant (Caki-2/VBL) or doxorubicin-resistant (Caki-2/DOX) was constructed, respectively. **a** The expressions of miR-210-3p in the RCC cells were determined by qRT-PCR. **b** The mRNA levels of ABCC1 expression in the RCC cells were assessed by qRT-PCR. **c** The protein levels of ABCC1 and MDR-1 in the RCC cells were detected by Western blot assays. *P < 0.05 vs. Caki-2

The RCC cell line Caki-2 with vinblastine-resistant (Caki-2/VBL) or doxorubicin-resistant (Caki-2/DOX) were constructed, respectively. The expressions of miR-210-3p (Fig. 1a), ABCC1 and MDR-1 protein (Fig. 1b, c) were determined. The results of qRT-PCR and Western blot assays showed that the expression of miR-210-3p was decreased and the levels of ABCC1 and MDR-1 were increased in Caki-2/DOX and Caki-2/VBL cells, compared to the RCC cell line Caki-2 (drug-sensitive cells).

### The expression of miR-210-3p could decline the drug resistance of RCC cells

**Fig. 2:**
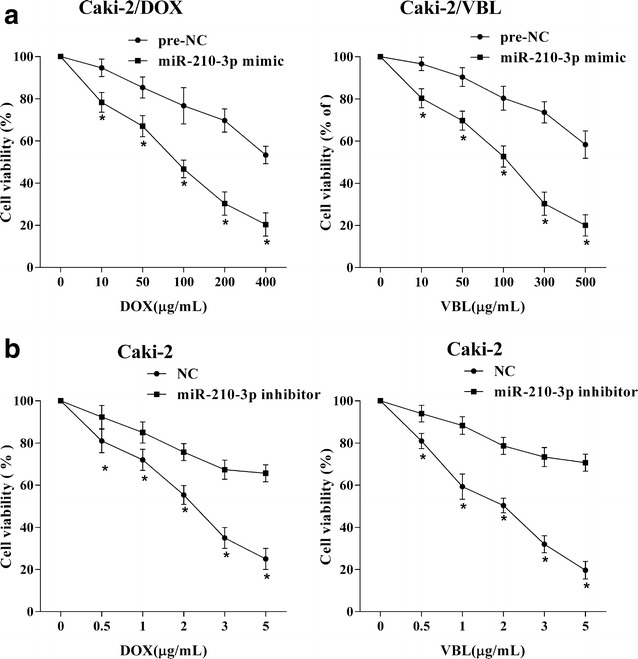
The influence of miR-210-3p on drug resistance of RCC cells. **a** After Caki-2/DOX and Caki-2/VBL cells were transfected with miR-210-3p mimic and then respectively treated with DOX (0, 10, 50, 100, 200, 400 μg/ml) and VBL (0, 10, 50, 100, 200, 300 μg/ml), followed by the cell viabilities were detected by MTT assays. *P < 0.05 vs. pre-NC. **b** After Caki-2 cells were transfected with miR-210-3p inhibitor and then respectively treated with DOX (0, 1, 2, 3, 4, 5 μg/ml) or VBL (0, 1, 2, 3, 4, 5 μg/ml), followed by the cell viabilities were detected by MTT assays. *P < 0.05 vs. NC

MiR-210-3p levels were up-regulated in Caki-2/DOX and Caki-2/VBL cells via transfection with miR-210-3p mimic, followed by treated with different concentration of DOX and VBL, respectively. The viabilities of Caki-2/DOX and Caki-2/VBL cells with miR-210-3p over-expression were declined, which suggested that up-regulation of miR-210-3p could elevate the drug-sensitivity of RCC cells (Fig. 2a). In addition, Caki-2 cells with miR-210-3p knockdown were treated with different concentrations of DOX or VBL. The increased viability of Caki-2 cells indicated that the drug-resistance of RCC cells was enhanced by miR-210-3p down-regulation (Fig. 2b).

### MiR-210-3p could regulate the ABCC1 expression

**Fig. 3:**
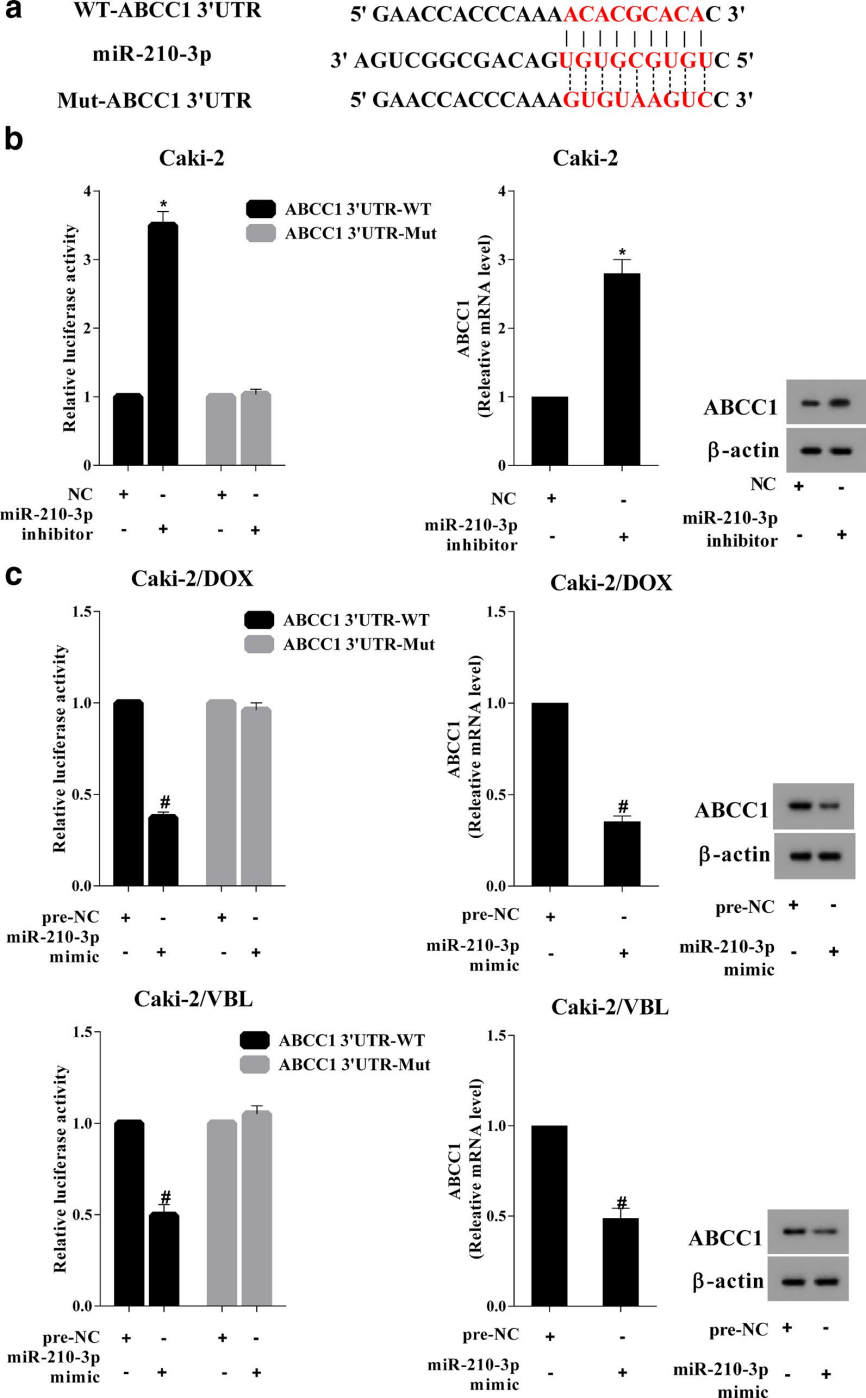
The mechanism of miR-210-3p regulating ABCC1. **a** The binding site of miR-210-3p on ABCC1 3′UTR was predicted by bioinformatics software. **b** In the Caki-2 cells, knockdown of miR-210-3p up-regulated the 3′UTR activity of wild type ABCC1 and the expression of ABCC1. **c** Over-expression of miR-210-3p inhibited the 3′UTR activity of wild type ABCC1 and the expression of ABCC1 in drug-resistant RCC cells. *P < 0.05 vs. NC, #P < 0.05 vs. pre-NC

For evaluating the regulatory relationship between miR-210-3p and ABCC1, Dual Luciferase assays were performed on RCC cell lines Caki-2, Caki-2/DOX and Caki-2/VBL. As shown in Fig. 3a, bioinformatics software predicted that miR-210-3p had the bind site on the 3′UTR of ABCC1. The luciferase activity of Caki-2 cells was increased by co-transfection with pGL3-ABCC1-WT and miR-210-3p inhibitor, and the luciferase activity of Caki-2 cells transfected with pGL3-ABCC1-Mut showed no difference after miR-210-3p knockdown. The expression of ABCC1 was up-regulated by miR-210-3p down-regulation at both mRNA and protein levels (Fig. 3b). MiR-210-3p mimic and pGL3-ABCC1-WT/pGL3-ABCC1-Mut were co-transfected into Caki-2/DOX and Caki-2/VBL cells, and the luciferase activities in Caki-2/DOX and Caki-2/VBL cells was reduced by co-transfection with pGL3-ABCC1-WT and miR-210-3p mimic. Meanwhile, the expression of ABCC1 was inhibited by miR-210-3p over-expression at both mRNA and protein levels (Fig. 3c).

### MiR-210-3p modulated MDR-1 expression and drug-resistance of RCC cells via ABCC1

**Fig. 4:**
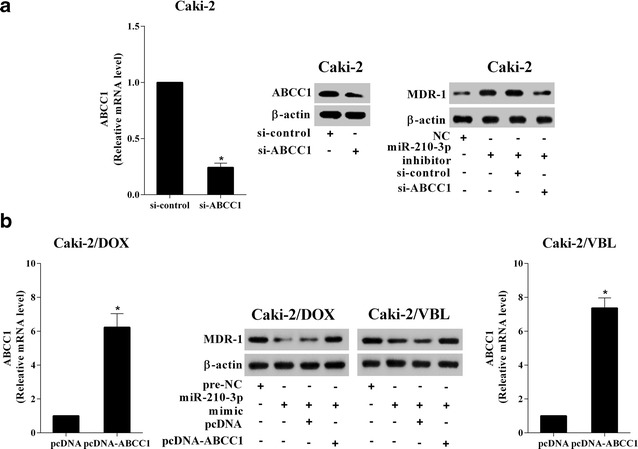
MiR-210-3p modulated MDR-1 expressions of RCC cells via ABCC1. **a** Caki-2 cells were transfected with si-ABCC1 or si-control, the knockdown efficiency of ABCC1 was detected using qRT-PCR and Western blot assays. Caki-2 cells were transfected with miR-210-3p inhibitor or miR-210-3p inhibitor + si-ABCC1, and then the levels of MDR-1 were assessed by Western blot assays. **b** Caki-2/DOX and Caki-2/VBL cells were transfected with pcDNA-ABCC1 or pcDNA, the overexpression efficiency of ABCC1 was detected using qRT-PCR and Western blot assays. Caki-2/DOX and Caki-2/VBL cells were transfected with miR-210-3p mimic or miR-210-3p mimic + pcDNA-ABCC1, and then the levels of MDR-1 were assessed by Western blot assays. *P < 0.05 vs. si-control or pcDNA

**Fig. 5:**
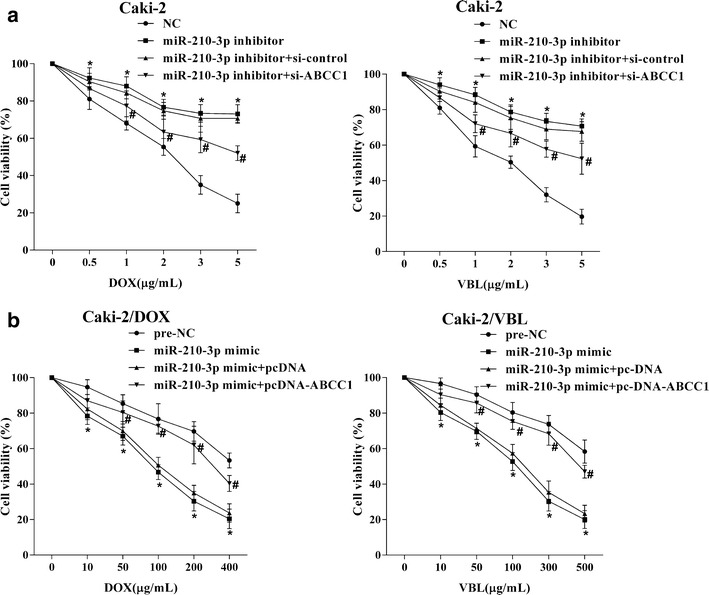
MiR-210-3p modulated drug-resistances of RCC cells via ABCC1. **a** Caki-2 cells were transfected with miR-210-3p inhibitor or miR-210-3p inhibitor + si-control, and then cell viabilities were assessed by MTT assays. **b** Caki-2/DOX and Caki-2/VBL cells were transfected with miR-210-3p mimic or miR-210-3p mimic + pcDNA-ABCC1, then cell viabilities were assessed by MTT assays. *P < 0.05 vs. NC or pre-NC,^#^P < 0.05 vs. miR-210-3p inhibitor + si-control or miR-210-3p mimic + pcDNA

After the drug-sensitive RCC cell line Caki-2 were transfected with miR-210-3p inhibitor, the level of MDR-1 in Caki-2 cell was enhanced. Then knockdown of ABCC1 in Caki-2 cell transfected with miR-210-3p inhibitor could reverse the effect of miR-210-3p down-regulation on the MDR-1 regulation (Fig. 4a). Meanwhile, the enhanced cell viability and drug-resistance induced by miR-210-3p knockdown were also reversed by ABCC1 inhibition in Caki-2 cell treated with different concentration of DOX or VBL (Fig. 5a). Moreover, the expression of miR-210-3p was up-regulated in the drug-resistant RCC cell lines, which obviously inhibited the level of MDR-1 in Caki-2/DOX and Caki-2/VBL cells. Further, the decreased levels of MDR-1 expression induced by miR-210-3p over-expression were reversed by the up-regulation of ABCC1 in Caki-2/DOX and Caki-2/VBL cells (Fig. 4b). Similarly, ABCC1 over-expression could reverse the miR-210-3p over-expression-induced the decrease of cell viability and drug-resistance in Caki-2/DOX and Caki-2/VBL cells treated with different concentrations of DOX or VBL (Fig. 5b).

### MiR-210-3p promoted the drug-sensitivity of RCC in mice

**Fig. 6:**
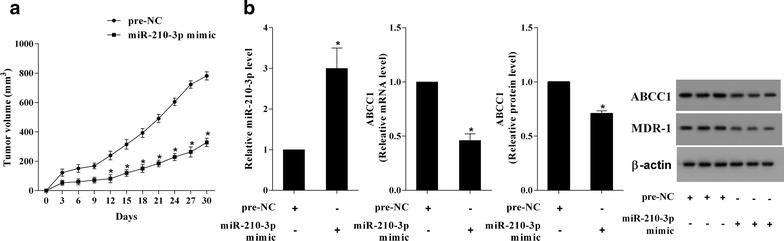
MiR-210-3p increased the drug-sensitivity of RCC. DOX was used to inject into the mice injected by Caki-2/DOX cells with/without miR-210-3p over-expression. **a** The tumor volume was markly reduced in the mice of miR-210-3p mimic group. **b** The levels of ABCC1 and MDR-1 were also declined in the mice of miR-210-3p mimic group. *P < 0.05 vs. pre-NC

**Fig. 7:**
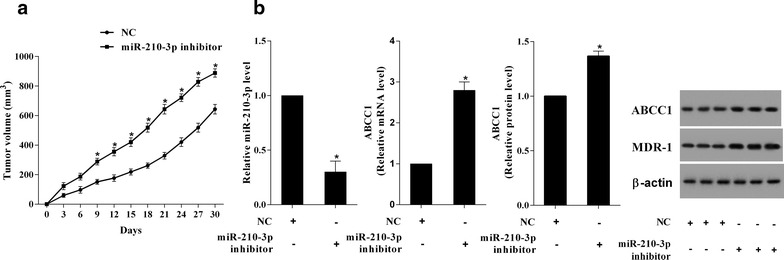
MiR-210-3p decreased the drug resistance of RCC. DOX was used to inject into the mice injected by Caki-2 cells with miR-210-3p inhibition. **a** The tumor volume was markly enhanced in the mice of miR-210-3p inhibitor group. **b** The levels of ABCC1 and MDR-1 were also enhanced in the mice of miR-210-3p inhibitor group. *P < 0.05 vs. NC

DOX was used to inject into the mice injected by Caki-2/DOX cells with/without miR-210-3p over-expression. The tumor volume was markly reduced in the mice of miR-210-3p mimic group, which exhibited that the miR-210-3p effectively enhanced the DOX-sensitivity of RCC to inhibit the growth of tumor (Fig. 6a). The levels of ABCC1 and MDR-1 were also declined in the mice of miR-210-3p mimic group (Fig. 6b). On the other hand, DOX was used to inject into the mice injected by Caki-2 cells with/without miR-210-3p inhibition. The DOX-resistance of RCC enhanced the speed of tumor growth in the mice of miR-210-3p inhibitor group (Fig. 7a). The levels of ABCC1 and MDR-1 were also remarkably elevated in the mice of miR-210-3p inhibitor group (Fig. 7b).

## Discussion

Chemotherapeutic unresponsiveness, metastatic spread and recurrence of RCC are mainly resulted from MDR. MiR-210-3p has been detected to be differently expressed between the drug-resistant and drug-sensitive RCC cells and involved in the drug-sensitivity of RCC cells. In our study, we focused on the role of miR-210-3p in the occurrence of RCC drug-resistance and further explored its underlying mechanism.

ATP-binding cassette transporter super-family has vital effect on MDR in cancer, which is reported to a main leading cause of chemotherapeutic failure in various cancers through regulating the efflux of chemotherapeutic drugs [15–18]. Previous reports have exhibited ABCC1 to be up-regulated in colorectal cancer [19], lung cancer [20], and breast cancer [21]. MDR-1 serving as a protein scavenger also can capture and transport various chemotherapeutic agents out of cells [22]. MDR-1 has been reported to be up-regulated in various chemotherapeutic-resistant cancer cell lines [23] and also be over-expressed in RCC patients [6]. The emergence of chemo-refractory with MDR greatly limits the efficacy and application of broad-spectrum conventional tumor chemotherapeutics [24]. In our study, we also observed the up-regulation of ABCC1 and MDR-1 in DOX-resistant and VBL-resistant RCC cells.

Emerging researches have indicated that several miRNAs were significantly associated to the recurrence and survival of patients with RCC and might act as biomarkers for the diagnosis of RCC patients with high risk in early recurrence phase after surgical resection in kidney [25]. In like manner, miRNAs promoted the phenotype of drug-resistant and the survival of tumor cell via directly targeting MDR family members to regulate the multi-drug resistance. MiR-210-3p was reported to highly express in clinical ccRCC specimens (compared to adjacent non-cancerous tissues) and RCC cell lines 786-o, A498 and Caki-2 (compared to normal kidney cells). However, Yoshino et al. reported that the higher expression of miR210-3p found in the ccRCC clinical samples and the cell lines was probably inhibitory to tumor progression, as shown by an accelerated cell invasiveness and an increased number of colonies in the miR-210-3p-depleted cells in comparison to the controls [11]. In the present study, the expression of miR-210-3p was decreased in Caki-2/DOX and Caki-2/VBL cells, compared to the RCC cell line Caki-2, suggesting the correlativity between miR-210-3p and drug-resistance of RCC cells. We further confirmed that miR-210-3p improved drug-sensitivity of RCC cells through inhibiting ABCC1. And up-regulation of miR-210-3p could decrease the drug-resistance and the levels of ABCC1 and MDR-1 in drug-resistant RCC cells. Hence, we confirmed that miR-210-3p mediated multi-drug resistance of RCC cells via binding with ABCC1. Moreover, miR-210-3p improved drug-sensitivity of RCC cells through inhibiting ABCC1. We identified the existence of miR-210-3p/ABCC1 axis in multidrug resistance of RCC cells, which also were proved in vivo.

In conclusion, we confirmed that down-regulation of miR-210-3p increased ABCC1 expression, thereby enhancing the MRP-1-mediated multidrug resistance of RCC cells, as shown by an increase in MDR1 expression and in cell viability with DOX or VBL treatment.
